# Public trust in AI-enabled telemedicine: affective, cognitive, and structural dimensions insights from multi-platform big data analytics

**DOI:** 10.3389/fmedt.2026.1789495

**Published:** 2026-06-02

**Authors:** Faisal Binsar, Mohammad Hamsal, Erwin Tenggono, Maman Abdurohman, Ridha Hanafi, Indra Wahyudi

**Affiliations:** 1 Management Department, Binus Online Learning, Bina Nusantara University, Jakarta, Indonesia; 2 Management Department, BINUS Business School Doctor of Research in Management, Bina Nusantara University, Jakarta, Indonesia; 3 Faculty of Economics and Business, Universitas Pelita Harapan, Tangerang, Indonesia; 4 Faculty of Economics and Business, Universitas Indonesia, Jakarta, Indonesia; 5 School of Computing, Telkom University, Bandung, Indonesia; 6 Information System Department, Faculty of Industrial Engineering, Telkom University, Bandung, Indonesia; 7 Faculty of Naval Architecture, Politeknik Negeri Bengkalis, Bengkalis, Indonesia

**Keywords:** AI-enabled telemedicine, big data analytics, digital health, health informatics, public trust, sentiment analysis

## Abstract

Artificial intelligence (AI) is transforming healthcare delivery, with telemedicine emerging as one of its most viable and socially impactful applications, raising important questions about how public trust is formed and sustained in AI-enabled healthcare environments. Yet, despite rapid technological adoption, public trust in AI-enabled telemedicine remains insufficiently understood, particularly across the diverse online environments where perceptions are collectively shaped. This study employs a sociotechnical, multi-platform big data analytics approach to quantify and interpret public trust in large-scale digital discourse. A total of 25,396 online mentions collected between 14 September and 14 October 2025 from news, social media, blogs, video, and web platforms were analyzed using a hybrid framework combining lexicon-based sentiment analysis (*VADER*), transformer-based contextual modeling (*RoBERTa*), and topic modeling (*BERTopic*). The results indicate a consistently positive sentiment orientation (*aggregate Trust Index = +0.84*), suggesting a favorable public outlook toward AI-enabled telemedicine during the observation period. Affective trust is more prominent in visual and community-based media, while cognitive and structural trust are more salient in institutional and professional contexts. These results inform an integrated conceptual model of public trust formation in AI-enabled telemedicine, conceptualizing trust as a multidimensional sociotechnical construct. Rather than introducing a novel methodology, this study demonstrates the integrative application of established computational techniques to capture public sentiment at scale. These insights provide practical guidance for healthcare providers, policymakers, and AI developers to design more transparent, patient-centered, and trustworthy telemedicine systems.

## 1 Introduction

The rapid integration of artificial intelligence (AI) into healthcare systems has transformed the practice of medicine (1), enabling data-driven diagnostics (2), personalized treatment (3), and remote patient monitoring on an unprecedented scale (4). Among the most profound manifestations of this transformation is the emergence of AI-enabled telemedicine (5), which combines intelligent automation with digital communication technologies to deliver healthcare beyond traditional clinical boundaries. During and after the COVID-19 pandemic, telemedicine shifted from a supplementary service to a mainstream mode of care, supported by advances in natural language processing, machine learning, and medical imaging. As healthcare delivery becomes increasingly mediated by algorithms, the question of public trust, in both the technology and the institutions deploying it, has become a defining determinant of sustainable digital health adoption (6).

Despite widespread enthusiasm for the efficiency and accessibility that AI-driven telehealth promises, public trust remains a fragile and context-dependent construct (7). Trust in this domain is not monolithic, it involves a dynamic interplay of *affective trust* (emotional confidence and empathy) (8, 9), *cognitive trust* (perceived competence and reliability) (10), and *structural trust* (confidence in institutional and technical systems) (11, 12). Previous studies have examined user acceptance of telemedicine or ethical perceptions of AI separately, yet few have systematically investigated how trust emerges, fluctuates, and is discursively constructed across the complex ecosystem of digital media where public opinion is formed. Moreover, most existing research relies on surveys or small-scale content analyses, offering limited generalizability in the era of large-scale, real-time online communication. In this context, while the present study utilizes sentiment analysis as a quantitative proxy, it is important to distinguish between sentiment and trust, as sentiment reflects emotional valence whereas trust encompasses broader cognitive, affective, and institutional dimensions. Therefore, any operationalisation of trust based on sentiment should be interpreted as indicative rather than definitive. In this study, the Trust Index is operationalized as a sentiment-based metric ranging from −1 (negative orientation) to +1 (positive orientation), representing the net balance of positive and negative expressions in the data.

To address this gap, the present study introduces a multi-platform big data analytics approach to quantifying public trust in AI-enabled telemedicine. This approach leverages large-scale digital trace data, which enables broad coverage of public discourse; however, reliance on a single commercial aggregation platform may introduce limitations related to data coverage, filtering mechanisms, and potential selection bias. Drawing on large-scale multi-platform online data from sources such as news portals, social media, blogs, forums, and video platforms, the study employs a hybrid sentiment–topic analytical framework that integrates lexicon-based sentiment detection (VADER) (13), transformer-based contextual classification (RoBERTa) (14), and topic modeling (BERTopic), a transformer-based approach that identifies semantically coherent themes in large textual datasets (15). This methodological synthesis enables a nuanced examination of both surface-level sentiment polarity and the deeper semantic structures that shape public attitudes toward digital healthcare innovations. Furthermore, the use of trust-related keywords in the data collection process may pre-condition the dataset toward trust-oriented discourse, potentially influencing the observed sentiment distribution.

The study advances two principal objectives. First, it seeks to quantify the intensity and direction of public trust in AI-enabled telemedicine across multiple digital environments. Second, it aims to uncover the underlying thematic and contextual dimensions through which trust and skepticism are articulated in public discourse. By linking sentiment dynamics with thematic structures, the research moves beyond polarity metrics to reveal how trust is embedded within narratives of technological efficacy, ethical responsibility, and patient-centered care. This analytical strategy aligns with emerging research in computational social science and health informatics that emphasizes data-driven epistemologies for understanding complex socio-technical phenomena (16, 17), while remaining primarily exploratory and descriptive in nature rather than inferential or causal. In line with this positioning, the proposed model should be understood as an exploratory and interpretative framework derived from observed patterns in the data, rather than a fully validated theoretical construct.

Ultimately, this study contributes primarily through an integrative application of established computational techniques to the growing literature on digital trust and AI in healthcare. Methodologically, it demonstrates how large-scale, heterogeneous online data can be transformed into indicative insights of collective perception using hybrid natural language analytics. Theoretically, it explores an integrated model of public trust formation that situates AI-driven telemedicine within the interplay of infrastructure, institutions, and individual experiences, with interpretations closely aligned to the empirical patterns observed in the data. In doing so, the study provides an exploratory perspective on AI ethics and telehealth acceptance by framing trust not merely as a psychological state but as a sociotechnical process of meaning-making, continually negotiated across diverse digital publics.

## 2 Materials and methods

This study utilizes publicly available online data and does not involve human subjects, personal identifiers, or private information. All data were collected and analyzed in accordance with ethical standards for digital research, ensuring that no individual users can be identified. Therefore, formal ethical approval was not required.

### 2.1 Data and scope

Data for this study were collected from Brand24, a commercial social listening and online analytics platform that aggregates publicly accessible web content across multiple digital ecosystems. However, as with most social listening tools, its coverage does not encompass the entire internet and is limited to indexed and publicly available sources. Content from private accounts, closed networks, restricted-access platforms, and certain region-specific or low-visibility websites may not be captured, which may affect the completeness and representativeness of the dataset. In addition, data coverage is influenced by proprietary crawling, indexing, and filtering mechanisms, which may introduce constraints on representativeness and reproducibility. The observation period spanned 14 September to 14 October 2025, corresponding to a phase of active public discussion surrounding the role of artificial intelligence (AI) in telemedicine and digital healthcare delivery. Only English-language content was included in the dataset to ensure consistency in sentiment analysis and compatibility with the applied natural language processing models.

A total of 25,396 online mentions were obtained from eight platform categories: news portals (online journalistic media), organizational or informational websites (non-news web domains), video platforms (e.g., YouTube), social media platforms (including X/Twitter and TikTok), blogs, and podcasts. These categories were defined to minimize overlap by distinguishing between editorial news content and general web-based informational sources. The inclusion of multiple platform categories aims to enhance diversity of discourse; however, differences in platform accessibility, API constraints, and content visibility may still influence the composition of the dataset. Data acquisition employed the Boolean query:

(“telemedicine” OR “telenursing” OR “telehealth”) AND (“AI trust” OR “Artificial Intelligence trust”)

The query design ensured comprehensive semantic coverage of AI-assisted remote healthcare discussions, including both professional and layperson-generated discourse. However, the inclusion of explicit trust-related keywords in the query may partially pre-condition the dataset toward trust-oriented narratives, potentially influencing the distribution of sentiment polarity and thematic emphasis.

Each record in the dataset contained the following fields: *title or headline, excerpt/comment text, source domain, follower or visit count, influence score, posting date, and platform*. The influence score represents a platform-derived metric estimating the potential visibility and engagement level of each mention, based on factors such as source reach and audience size, and was used for descriptive comparison across platforms. These variables enabled multi-level quantitative and semantic analysis, spanning from sentiment polarity and topical structure to comparative patterns across media types.

### 2.2 Data preprocessing

A rigorous preprocessing pipeline was implemented to ensure analytical consistency and noise reduction. Duplicates, commercial advertisements and non-relevant content were removed based on predefined criteria, including repetitive promotional language, excessive use of hyperlinks, lack of substantive informational content, and clear indicators of automated or spam-like posting behavior. *Text Normalization* using the *spaCy library*, texts were converted to lowercase, tokenized, stripped of stop words and punctuation, and lemmatized to their root forms. This process standardized morphological variations (e.g., *patients → patient*), improving consistency in lexical feature extraction. Residual placeholders and system-generated artifacts, such as “[…]”, “Neutral […]”, or “…” appearing at the beginning of excerpts, were systematically removed using regular expressions.

### 2.3 Analytical procedure

The analytical workflow integrated three major stages: (1) *descriptive and comparative analytics,* (2) *sentiment quantification*, and (3) *topic modeling*. This analytical design is primarily descriptive and exploratory, aiming to identify patterns and structures within large-scale textual data rather than to establish causal relationships or predictive inference. All procedures were implemented using Python 3.11, leveraging *pandas*, *vaderSentiment*, *transformers*, *BERTopic*, *matplotlib*, and *seaborn* (18). Prior to sentiment analysis, the dataset was filtered to include only English-language content to ensure compatibility with both VADER and RoBERTa models.

Quantitative summaries were first produced to describe the overall data structure and cross-platform variation. Descriptive statistics, such as total records, proportional shares, mean influence scores, and temporal frequencies, were computed to characterize the magnitude and spread of online discourse across platforms. Comparative analytics were then used to evaluate differences among platforms in terms of content volume, estimated audience reach, and influence metrics. All results were visualized using *matplotlib* and *seaborn* to generate frequency distributions, percentage bar charts, and daily mention–reach time series. This descriptive foundation provided the empirical context for subsequent analyses of sentiment polarity and thematic structure.

Sentiment polarity was operationalized as a quantitative proxy for public trust orientation toward AI-enabled telemedicine. This operationalization is grounded in prior computational social science research, where sentiment polarity is commonly used as a scalable approximation of attitudinal orientation in large-scale textual datasets, while acknowledging that trust itself is inherently multidimensional. While sentiment polarity provides a scalable and data-driven indicator of public attitudes, it does not fully capture the multidimensional construct of trust, which encompasses cognitive, affective, and institutional components. Therefore, the Trust Index should be interpreted as an indicative proxy of public sentiment orientation, rather than a direct or fully validated measure of trust. This operationalisation follows prior computational social science approaches that use sentiment as an approximation of attitudinal constructs in large-scale textual data. A hybrid two-layer sentiment architecture was applied:

1. *Lexicon-based Layer*: *The Valence Aware Dictionary and sEntiment Reasoner (VADER)*, a rule-based sentiment analysis model developed by Hutto & Gilbert (19), was employed to calculate an initial polarity score for each text. VADER is particularly effective for social-media-style language, as it integrates grammatical heuristics (e.g., capitalization, punctuation intensity, degree modifiers) and a sentiment lexicon calibrated for microtext. Each record received a *compound score* between −1 (negative) and +1 (positive), with thresholds at ±0.05 for neutral classification.

Formally:

$$S_{v}=compound(t)\in [-1,\;+1]$$

where *t* denotes the tokenized comment or excerpt.

(1) *Transformer-based Layer (RoBERTa)*: To capture contextual subtleties and domain-specific semantics in healthcare communication, a RoBERTa-base model (14) fine-tuned on medical and health-related corpora was applied. This step adjusted for cases where lexicon-based scores may misclassify technically phrased but sentiment-bearing statements (e.g., “AI triage systems outperform physicians” → positive intent, neutral lexicon).
(2) Fusion and Classification: The two layers were integrated through weighted averaging, producing a final polarity label (*positive*, *neutral*, *negative*). The Trust Index for each platform was computed as:
  $$T_{i}=\;\frac{N_{\;pos}-N_{neg}}{N_{total}}$$

where *T_i_* denotes the net trust score for platform *i*. *N_pos_*, *N_neg_*, and *N_total_* represent the number of positive, negative, and total mentions, respectively. This index ranges from −1 (complete distrust) to +1 (complete trust), allowing direct cross-platform comparison.

To uncover recurrent themes and discursive structures, *BERTopic* (15) was applied. This algorithm combines *transformer-based embeddings* (via *Sentence-BERT*) with *class-based TF–IDF* (c-TF–IDF) to identify semantically coherent clusters. The coherence score *C_v_* was used to assess topic interpretability. The resulting clusters captured salient domains such as *data privacy*, *diagnostic accuracy*, *accessibility*, and *ethical concerns in AI healthcare*.

**Figure 1:**
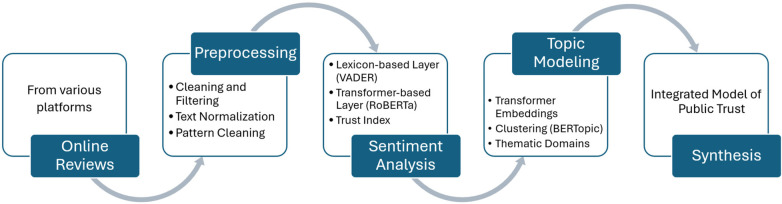
Integrated analytical framework.

Overall, the integrated analytical framework of this study is illustrated in Figure 1, which visualizes the end-to-end workflow from data acquisition to semantic insight extraction. This framework consolidates all methodological components previously described, including multi-platform online data collection and preprocessing, hybrid sentiment analysis, and transformer-based topic modeling, into a unified and coherent analytical system. It represents a Big Data analytics framework applied to examine public trust in AI-enabled telemedicine, combining both quantitative and semantic dimensions of digital discourse across heterogeneous media environments. It should be noted that the observation period is limited to one month, which captures a specific temporal snapshot of public discourse. As public trust in AI-enabled telemedicine is dynamic and context-dependent, the findings should be interpreted within this temporal scope and should not be generalized beyond the observed period.

## 3 Results

### 3.1 Descriptive analytics

**Figure 2:**
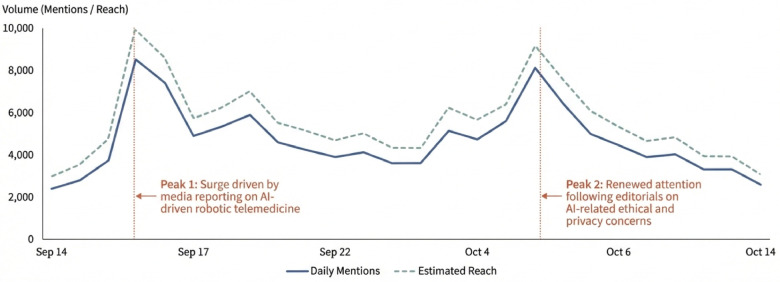
Daily mentions and estimated reach of online discussions.

A total of 25,396 online mentions concerning *AI-enabled telemedicine* were captured between 14 September and 14 October 2025, covering multiple public communication channels. As shown in Figure 2, the temporal trend of daily *mentions* (solid blue line) and *reach* (dashed green line) reveals a distinctly oscillatory pattern with periodic peaks and troughs throughout the observation window.

Two major spikes were observed: (1) around 15–17 September 2025, coinciding with extensive media reporting and video releases on *AI-driven robotic telemedicine*; and (2) between 4 and 6 October 2025, corresponding to renewed attention following the publication of editorials on *AI-related ethical and privacy concerns* in digital health. Across the 30-day period, daily mention volumes fluctuated between 600 and 1,900, while estimated audience reach ranged from 1 to 6 million users, indicating a strong temporal coupling between discussion intensity and information diffusion. The synchronic movement of both variables suggests a possible association between message volume and amplification through high-visibility news outlets and cross-platform sharing mechanisms, although causal relationships cannot be established within this descriptive analysis.

**Figure 3:**
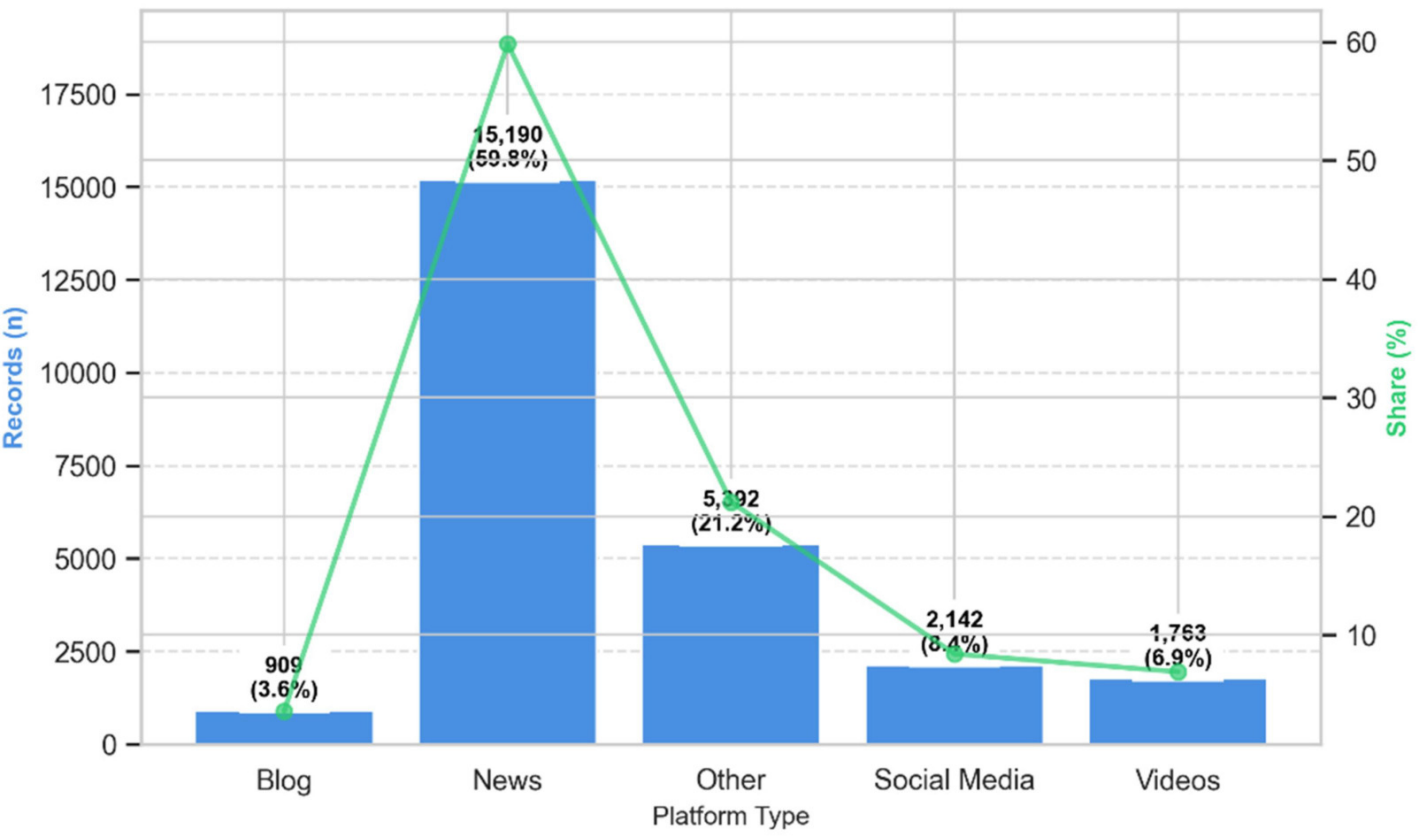
Distribution of collected data by platform.

Platform-level distribution further demonstrates the asymmetry of information production (Figure 3). News media overwhelmingly dominated the conversation, contributing 59.8% of all records (*n* = 15,190). This underscores the continued centrality of institutional journalism in shaping the public narrative on AI-based healthcare systems. The “Other” category, including aggregated health portals and organizational websites, accounted for 21.2%, while social-media channels (e.g., X/Twitter, TikTok, LinkedIn) represented 8.4%, and video platforms (e.g., YouTube) contributed 6.9%. Blogs formed a smaller yet thematically rich subset (3.6%) dominated by experiential and reflective commentaries. It should also be noted that these patterns reflect a limited one-month observation period and may not capture longer-term dynamics of public trust, which are inherently temporal and context-dependent.

### 3.2 Sentiment analysis and trust quantification

A detailed sentiment quantification was conducted to assess the polarity and intensity of public trust toward AI-enabled telemedicine across digital platforms. Using the hybrid sentiment framework that combines the lexicon-based VADER model and the contextual RoBERTa classifier, each textual record was categorized as *positive*, *neutral*, or *negative*, and a platform-specific Trust Index (*Ti*) was computed to represent the net sentiment orientation. The use of a hybrid two-layer sentiment architecture was not intended to increase computational complexity but to ensure analytical robustness. The VADER layer effectively captured affective trust dynamics across user-generated platforms, while the RoBERTa layer validated contextual consistency in professional and news discourse. The convergence of both layers provides indicative support for the consistency of the computed Trust Index, although it should not be interpreted as a formal validation of the construct.

**Figure 4:**
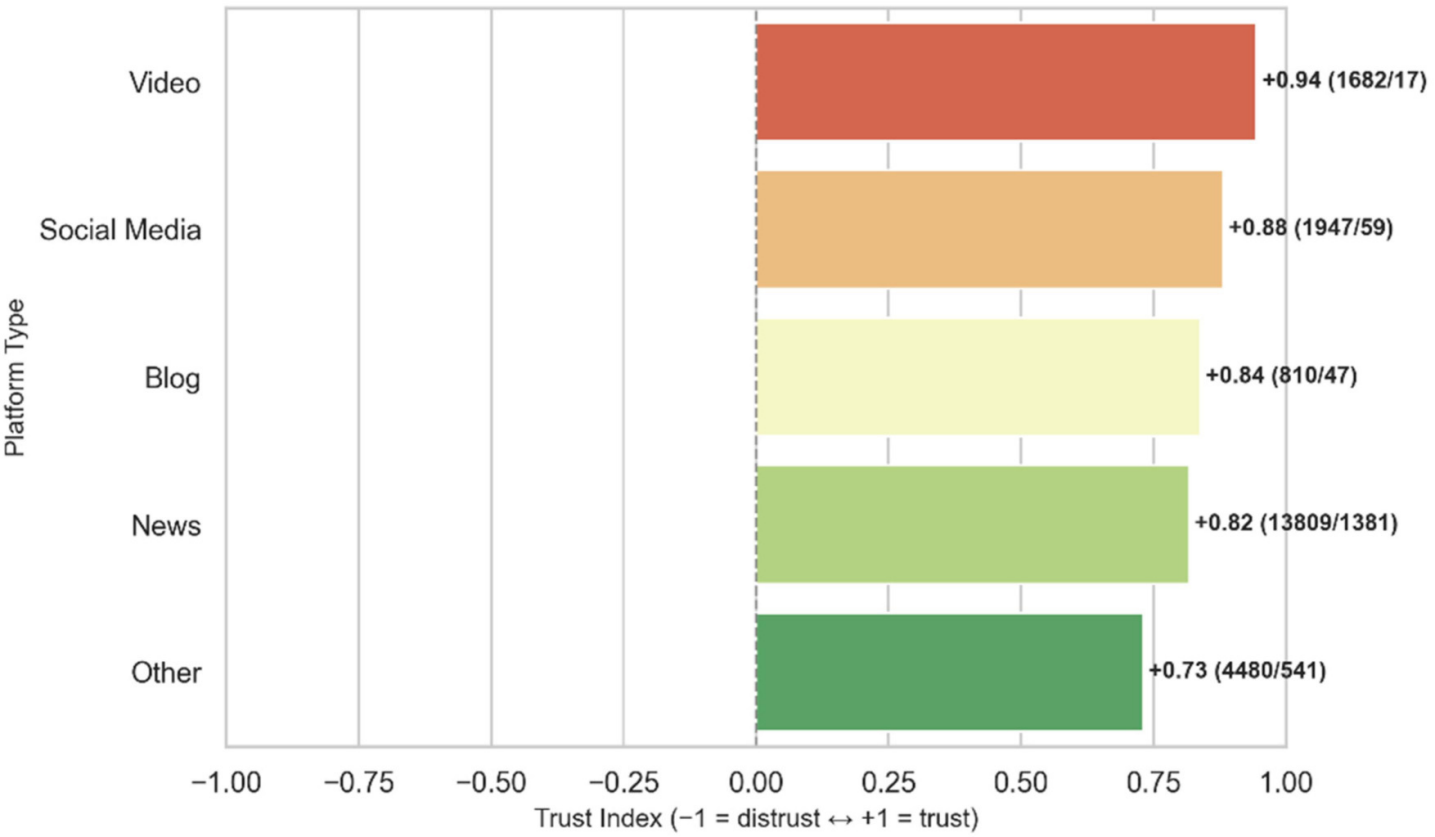
Trust index per platform (AI-enabled telemedicine).

Despite quantitative dominance by news content, the *Trust Index* analysis revealed high levels of positive orientation across all platform categories (Figure 4), with all indices exceeding +0.70, indicating overall optimism and favorable attitudes toward the integration of AI technologies in telemedical services. However, the consistently high positive sentiment observed across platforms may partly reflect underlying data or methodological biases, including query design and platform-specific content dynamics, and should therefore be interpreted with caution.

Among the platforms, video-based media exhibited the highest trust level (*Ti* *=* *0.945*), which may be associated with the persuasive characteristics of audiovisual narratives and experiential demonstrations that can enhance perceived transparency and reliability of AI-driven healthcare tools. Social media content followed with a trust index of *0.882*, which may reflect the role of peer-to-peer communication and user-generated testimonials in shaping confidence and engagement within participatory digital spaces. Blogs (*Ti* *=* *0.839*) and news portals (*Ti* *=* *0.818*) also showed substantial positive sentiment, although these sources tended to mix informative and critical discourses, balancing enthusiasm with caution on issues such as data privacy, professional accountability, and algorithmic transparency. Meanwhile, the Other category (aggregated professional and institutional sites) displayed a moderately positive trust index (*Ti* *=* *0.731*), indicating that while institutional narratives recognize the strategic value of AI, they often emphasize implementation risks and regulatory considerations.

Taken together, the descriptive statistics indicate a predominantly high-trust and media-amplified pattern of public discourse for AI-enabled telemedicine during the study period. While traditional media dominated content volume, the highest expressions of trust originated from participatory and visual platforms, which may reflect the influence of experiential and demonstrative communication formats on public perceptions of healthcare technologies.

Cross-platform comparison suggests that audience interactivity and narrative modality play significant roles in shaping public sentiment. Platforms allowing visual engagement and personal storytelling (e.g., YouTube, TikTok) demonstrated the highest emotional positivity, whereas text-dominant institutional media displayed more analytical and balanced tones. This pattern is consistent with prior findings suggesting that media richness and experiential framing may be associated with higher perceived credibility and emotional resonance of emerging technologies. Furthermore, despite minor fluctuations across platforms, negative sentiment remained consistently low, largely centered on concerns over data security, diagnostic accountability, and potential depersonalization of care.

Overall, the sentiment analysis highlights a high-trust and positively oriented digital discourse toward AI-enabled telemedicine during the observation period. The dominance of positive sentiment across heterogeneous media ecosystems suggests a tendency toward increasing normalization and acceptance of AI-assisted healthcare within the observed digital discourse. These findings serve as a descriptive basis for the subsequent topic modeling, which explores the thematic structures and contextual narratives underlying these trust dynamics.

### 3.3 Topic modeling and thematic insights

To complement the sentiment quantification results, topic modeling was conducted using the BERTopic algorithm (20) to uncover the latent semantic structures underlying public discourse on AI-enabled telemedicine. BERTopic was selected due to its ability to integrate transformer-based embeddings with density-based clustering, enabling the identification of coherent and contextually meaningful topics across large-scale, heterogeneous textual data. After model optimization and topic reduction, ten distinct and interpretable topics were identified, representing the dominant domains of discussion across news outlets, social media platforms, blogs, video content, and other web sources.

Each topic was characterized by a set of representative keywords and contextual interpretations, as summarized in Table 1. The results indicate that online discourse is heavily dominated by general discussions of AI-driven telemedicine practices and clinical integration (Topic 0), followed by patient-centered and mental health–related narratives (Topic 1). Together, these two topics account for the majority of total mentions, highlighting that public engagement is primarily oriented toward the practical application of AI in healthcare delivery and its perceived impact on patient well-being.

**Table 1:** Top 10 topics identified by BERTopic.

Topic	Count	Representative Keywords	Initial Interpretation
0	12,024	Telemedicine, AI, healthcare, system, doctor	Core discourse on AI-driven telemedicine practices and integration into clinical workflows.
1	10,607	Telehealth, mental, medicare, patient	Mental health and patient experience in telehealth systems, including access and support.
2	720	Clinic, appealed, not, was, tried	Clinical case discussions, patient appeals, and practical experiences of telemedicine delivery.
3	627	Companies, telehealth, letters, licensing	Industry actors, licensing processes, and corporate involvement in telehealth expansion.
4	280	5G, latency, IoT, real-time, infrastructure	Technological infrastructure and digital connectivity enabling remote care (5G, IoT).
5	279	Insurance, options, fay, coverage	Health insurance, cost coverage, and policy options for telemedicine.
6	232	Hims, Hers, telehealth, brand	Commercial telehealth brands and marketing discourse in digital health.
7	221	Eye, cybersight, training, surgeon	Tele-ophthalmology and professional capacity building for remote diagnostics.
8	209	Veterinary, pet, treatment, service	Tele-veterinary practices as extensions of telemedicine into non-human health domains.
9	197	Various, emerging, trends, start-ups	Emerging and miscellaneous discussions on new AI-enabled healthcare innovations.

Beyond these dominant themes, several lower-frequency but analytically significant topics emerged. Technical infrastructure discussions (Topic 4) emphasize the role of 5G, IoT, latency, and real-time connectivity as foundational enablers of remote care. Institutional and economic narratives are reflected in Topics 3, 5, and 6, which focus on corporate actors, licensing arrangements, insurance coverage, and the commercialization of telehealth services. Meanwhile, specialized applications such as tele-ophthalmology (Topic 7) and tele-veterinary medicine (Topic 8), along with emerging innovation-oriented discussions (Topic 9), illustrate the diversification and expansion of telemedicine beyond conventional patient–physician interactions.

While the ten-topic structure provides granular insights, interpreting trust-related dynamics at this level risks fragmentation. Therefore, to enhance analytical clarity and theoretical interpretability, the ten topics were systematically consolidated into five higher-order thematic clusters based on lexical similarity, discursive function, and shared semantic orientation, following established practices in health informatics and computational discourse analysis (21). This consolidation is presented in Table 2.

**Table 2:** Consolidation of 10 BERTopic topics into 5 thematic clusters.

Thematic Cluster	Constituent Topics	Combined Frequency	Conceptual Focus
1. AI-Driven Telemedicine Practices	0, 2	12,744	Core discussions of AI-based diagnostics, clinical integration, and operational efficiency in telemedicine.
2. Mental Health and Patient Experience	1	10,607	Patient-centered narratives emphasizing accessibility, emotional well-being, and remote mental health support.
3. Digital Infrastructure and Technical Enablers	4	280	Conversations highlighting technological foundations (5G, IoT, connectivity) of remote care delivery.
4. Industry and Policy Landscape	3, 5, 6	1,138	Economic and institutional aspects of telehealth, corporate actors, insurance, and regulatory mechanisms.
5. Specialized and Emerging Telehealth Domains	7, 8, 9	627	Niche applications such as tele-ophthalmology, tele-veterinary, and new AI-enabled healthcare startups.

The first cluster, AI-Driven Telemedicine Practices, combines Topics 0 and 2 and represents the operational core of public discourse. This cluster captures discussions surrounding AI-supported diagnostics, clinical workflows, and real-world patient interactions. Its substantial combined frequency underscores that public trust appears to be associated with perceptions of functional performance, clinical reliability, and seamless integration into healthcare routines. This aligns with prior findings that trust in AI systems is primarily shaped by their demonstrated usefulness and embedding within professional practice contexts (22).

The second cluster, Mental Health and Patient Experience, corresponds directly to Topic 1 and reflects a distinctly human-centered discourse. This domain emphasizes accessibility, emotional support, and patient engagement within telehealth environments, particularly in mental health contexts. The prominence of this cluster suggests that affective and experiential considerations may play an important role in shaping public trust, especially where AI-mediated care intersects with vulnerability, empathy, and continuity of care.

The Digital Infrastructure and Technical Enablers cluster (Topic 4), although smaller in volume, highlights the material and technological foundations of AI-enabled telemedicine. Discussions within this cluster foreground connectivity, system responsiveness, and infrastructural readiness, indicating that segments of public discourse reflect awareness of the dependency of telemedicine trust on stable and secure technical architectures. These narratives contribute to structural trust by emphasizing system dependability rather than direct user experience.

The Industry and Policy Landscape cluster consolidates Topics 3, 5, and 6, encompassing corporate involvement, insurance mechanisms, and regulatory frameworks. This cluster reflects an institutional discourse that situates AI-enabled telemedicine within broader governance and economic systems. Although less emotionally charged, these discussions may shape public perceptions of legitimacy, accountability, and sustainability, reinforcing trust at the organizational and systemic level.

Finally, the Specialized and Emerging Telehealth Domains cluster (Topics 7, 8, and 9) captures niche and forward-looking applications, including tele-ophthalmology, tele-veterinary services, and emerging AI healthcare innovations. Despite their lower frequencies, these topics are analytically significant as they signal an expanding conceptualization of telemedicine, suggesting that public trust is extending toward novel use cases and specialized domains beyond traditional healthcare boundaries.

**Figure 5:**
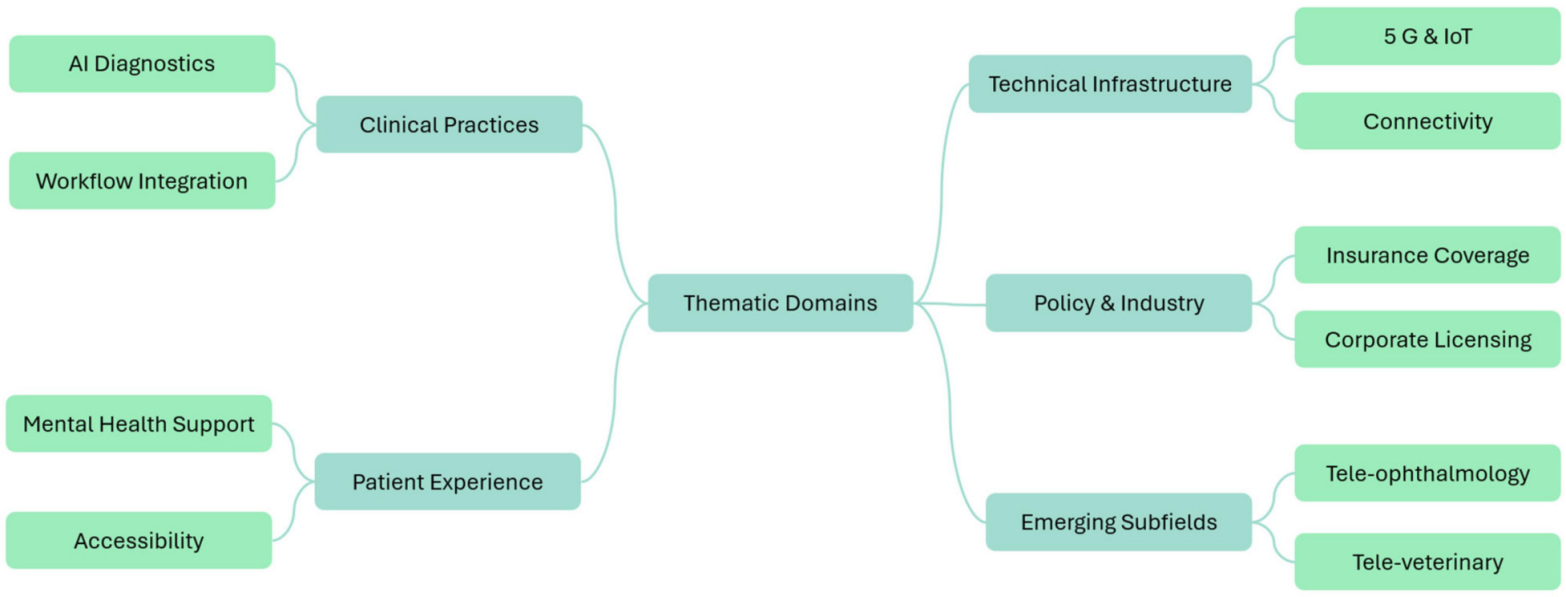
Thematic Domains.

These five thematic clusters are visually synthesized in Figure 5, which illustrates the hierarchical and relational structure of public discourse on AI-enabled telemedicine. The figure highlights how technical infrastructure, institutional arrangements, and specialized domains converge through AI-driven telemedicine practices to shape patient-centered experiences. Together, the thematic consolidation and visualization suggest that public discourse (and by extension, interpretations of public trust) may be shaped by the interaction of operational, experiential, technical, and institutional dimensions rather than from any single factor in isolation.

## 4 Discussion

The findings of this study provide an integrative perspective on how public trust in AI-enabled telemedicine is constructed, expressed, and differentiated across digital environments. By leveraging a large-scale, multi-platform dataset and a hybrid analytical pipeline combining sentiment quantification (VADER–RoBERTa) with topic modeling (BERTopic), this research elucidates both the *affective orientation* and *semantic structure* of public discourse (9, 10). The empirical results suggest that public attitudes toward AI-driven telemedicine were generally positive during the observation period but vary in their thematic depth, emotional tone, and platform-specific framing. These findings should be interpreted as context-specific observations derived from a limited temporal and methodological scope, rather than as broadly generalizable conclusions. The predominance of positive sentiment should also be critically considered, as it may partially reflect data selection effects, platform dynamics, or query-related biases rather than purely organic public attitudes.

### 4.1 Multidimensional nature of public trust

The findings indicate that public trust in AI-enabled telemedicine can be understood as a *multidimensional construct*, not merely an emotional reaction but a layered system of perceptions spanning cognitive, affective, and structural dimensions. It is important to note that the Trust Index used in this study represents a sentiment-based proxy and does not fully capture the multidimensional construct of trust, which includes cognitive, affective, and institutional components. The sentiment analysis demonstrated consistently positive trust indices across all platforms, with the highest trust observed in video-based and social media environments, where users engage through experiential narratives and peer-to-peer exchanges. These platforms appear to be associated with affective dimensions of trust, derived from personal and vicarious experiences that convey empathy and reliability. Conversely, the news and blog platforms exhibited more cognitive trust (23), reflecting a rational appraisal of AI's role in healthcare quality, ethics, and governance.

This distinction aligns with prior literature on digital trust formation, which highlights that experiential media tend to evoke emotion-driven trust, while informational media strengthen belief-based and institutional trust (24, 25).

### 4.2 Thematic structures and discourse ecologies

The ten topics extracted through BERTopic and subsequently consolidated into five macro-level thematic domains provide conceptual insight into the *discursive ecology* surrounding AI-enabled telemedicine. The largest cluster, *AI-driven telemedicine practices*, represents the epistemic core of public conversation, emphasizing clinical efficiency, diagnostic enhancement, and technological integration. This domain acts as a *semantic bridge* connecting the infrastructural, institutional, and patient-centered dimensions of trust.

The *mental health and patient experience* cluster underscores a critical human-centered orientation (26), suggesting that emotional safety, accessibility, and continuity of care remain at the heart of digital health acceptance. In parallel, the *digital infrastructure and technical enablers* domain, centered on discussions of 5G, IoT, and system reliability, reflects the emergence of structural trust, wherein confidence in system robustness translates into confidence in telemedicine as a whole.

The *industry and policy landscape* cluster reveals how corporate and institutional actors shape trust discourse (27), either reinforcing it through transparent regulation or weakening it through perceived commercialization. Finally, the *specialized and emerging telehealth domains* (e.g., tele-ophthalmology, tele-veterinary) highlight the diversification of AI-driven healthcare, expanding the notion of “telemedicine” beyond conventional clinical boundaries.

### 4.3 Integrative framework of trust formation

**Figure 6:**
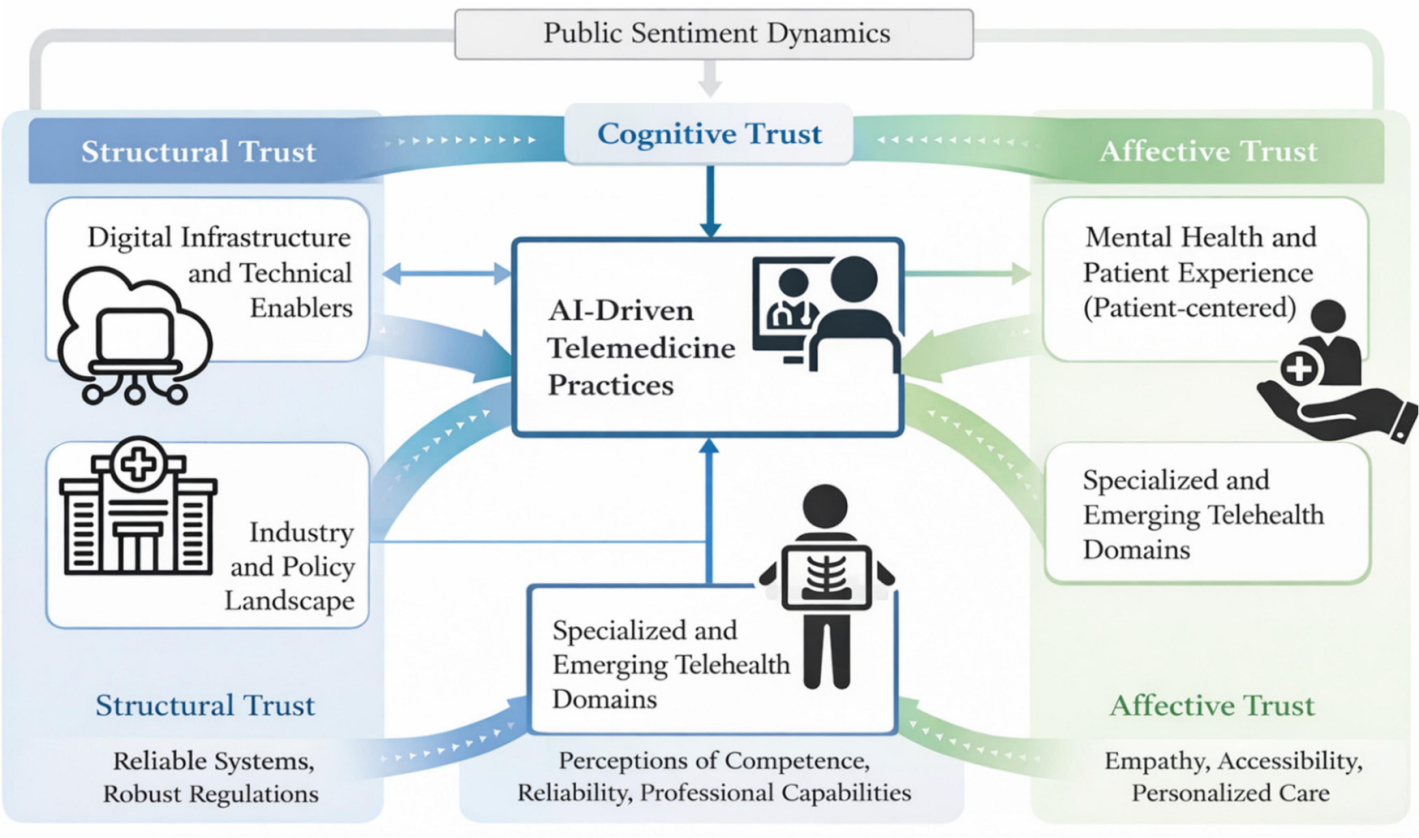
Integrated framework of public trust dynamics in AI-enabled telemedicine.

Synthesizing the empirical and analytical layers, this study tentatively conceptualizes public trust in AI-enabled telemedicine as an emergent and dynamic sociotechnical process shaped by interrelated technological, institutional, and experiential forces (Figure 6). The proposed framework illustrates how *Digital Infrastructure and Technical Enablers*, *Industry and Policy Landscapes*, and *Specialized and Emerging Telehealth Domains* converge in the operational core of *AI-Driven Telemedicine Practices*, which in turn shape and are shaped by *Patient-Centered Mental Health and Experience* (26). This directional flow represents not merely a linear pathway of influence but a recursive cycle of trust formation, continually negotiated through public discourse and collective sentiment online.

Within this architecture, trust dimensions manifest across distinct but interacting layers. *Structural trust* originates in the technological and institutional foundations (encompassing data security, algorithmic accountability, and regulatory legitimacy) that underpin perceptions of systemic reliability (11, 28). *Cognitive trust* emerges at the operational level, where users evaluate AI's competence, transparency, and fairness within telemedical practices (8, 10). *Affective trust*, by contrast, materializes within patient-centered experiences, grounded in empathy, perceived care, and emotional assurance fostered through mediated interactions (9). These dimensions operate synergistically, creating a vertical continuum from infrastructural assurance to experiential confidence. In the integrative framework (Figure 6), structural trust is rooted in the foundational domains of infrastructure and policy, cognitive trust materializes through operational telemedicine practices, and affective trust culminates in patient-centered experiences. Encircling these layers, public sentiment dynamics function as a continuous feedback loop, mediating how societal perceptions evolve and feed back into technological and institutional adaptation. The proposed framework should therefore be understood as an exploratory and interpretive model derived from observed patterns in the data, rather than a fully validated or generalizable theoretical construct.

Table 3 summarizes how the three trust dimensions identified in the integrative framework align with the empirical patterns observed across sentiment polarity, platform typology, and topic clusters. This mapping provides an interpretive bridge between data-driven analytics and the conceptual architecture of trust formation.

**Table 3:** Alignment between empirical findings and trust dimensions in AI-enabled telemedicine.

Trust Dimension	Conceptual Definition	Empirical Representation in Findings	Illustrative Evidence from Data
Structural Trust	Confidence grounded in institutional, regulatory, and technological reliability (11, 28)	Foundations of digital infrastructure, governance, and policy frameworks	Topics on *AI regulation*, *data security*, and *institutional legitimacy* prevalent in *News* and *Blogs*
Cognitive Trust	Rational belief in system competence, accuracy, and transparency (8, 10)	Evaluative discourse on AI-driven diagnostics, teleconsultation performance, and efficiency	Clusters around *AI accuracy*, *medical reliability*, and *clinical efficiency*
Affective Trust	Emotional assurance based on empathy, perceived care, and psychological comfort (8, 9, 25)	Patient-centered narratives and supportive communication across social and visual media	Positive sentiment in *Video* and *Social Media* platforms emphasizing *patient well-being* and *emotional support*

Encircling this layered structure are public sentiment dynamics, which function as the feedback mechanism that sustains or destabilizes trust over time. Through waves of online discourse, expressed in mentions, reach, and sentiment polarity, collective perceptions are continuously updated, influencing institutional responses, communication strategies, and technological adaptation. Hence, trust in AI-enabled telemedicine is not static but a reflexive system, co-constructed through the interplay of technical affordances, policy frameworks, and the emotional economies of digital publics.

The framework aligns with the sociotechnical systems perspective (29) and extends contemporary models of AI trust formation by embedding the affective and discursive dimensions often omitted from purely technical accounts. It therefore provides a holistic conceptualization of trust as both a structural condition and an evolving communicative process, highlighting how legitimacy and acceptance of AI-driven healthcare are continuously shaped within the hybrid ecology of infrastructure, institutions, and mediated human experience (30). As such, the model serves as a heuristic representation of trust dynamics that requires further empirical validation across different datasets, contexts, and methodological approaches.

### 4.4 Theoretical and practical implications

From a theoretical standpoint, the findings contribute to the literature on digital trust, AI ethics, and telehealth acceptance by demonstrating how sentiment polarity and thematic co-occurrence can serve as proxies for public trust metrics in large-scale data environments. The hybrid methodology employed here, combining lexicon-based and transformer-based sentiment analysis with unsupervised topic modeling, offers a scalable, reproducible approach to *computational trust analytics* (31).

This contributes to emerging “trust analytics” frameworks within AI governance research, providing empirical grounding to conceptual debates that often remain abstract.

Practically, the insights offer actionable implications for health policymakers, digital health developers, and AI ethics boards.

- For policymakers, the prevalence of positive but emotionally-driven trust underscores the need to reinforce cognitive trust through transparency, explainability, and ethical communication of AI capabilities.
- For telemedicine providers, emotional resonance and patient-centered narratives, particularly in visual and interactive platforms, represent crucial levers for trust-building.
- For AI system designers, the findings stress the importance of integrating *explainable AI (XAI)* elements that align with both affective and cognitive dimensions of trust formation.

### 4.5 Limitations and future directions

The reliance on a single commercial data aggregation platform (Brand24) introduces potential limitations related to data coverage, proprietary filtering mechanisms, and reproducibility, as the underlying data collection processes are not fully transparent. In addition, the use of trust-related keywords in the data collection query may have pre-conditioned the dataset toward trust-oriented discourse, potentially inflating the prevalence of positive sentiment. Furthermore, the one-month observation period captures only a temporal snapshot of public discourse and may not reflect longer-term fluctuations in trust, which are inherently dynamic and context-dependent.

More broadly, this study is limited by the use of sentiment analysis as a proxy for trust, which may oversimplify complex attitudinal constructs and overlook nuances such as ambivalence, skepticism, or conditional acceptance. Additionally, the descriptive and correlational nature of the analysis restricts the ability to infer causal relationships between discourse patterns and trust formation. These limitations highlight the need for complementary methodological approaches, including longitudinal, experimental, or mixed-method designs, to validate and extend the findings.

While the study provides a robust cross-platform analysis, it remains limited by its reliance on publicly available English-language content and keyword-based data retrieval. Non-textual modalities (e.g., images, videos) were not semantically analyzed, which may underrepresent multimodal trust cues prevalent in visual platforms such as TikTok or YouTube. Future work could employ multimodal sentiment models or cross-linguistic comparative frameworks to capture a more global trust ecology. Moreover, longitudinal analyses beyond a one-month observation period could reveal temporal fluctuations in public sentiment, particularly in response to major technological or policy events in digital healthcare.

## 5 Conclusion

This study provides an integrative and data-driven understanding of how public trust in AI-enabled telemedicine is constructed, expressed, and distributed across digital environments. By combining multi-platform big data analytics with a hybrid sentiment–topic modeling framework, the research suggests that public perception of AI in telehealth was generally positive during the study period. The consistent positive Trust Index observed across platforms suggests a favorable orientation during the study period, although this pattern should be interpreted cautiously given potential data and methodological limitations. Accordingly, the Trust Index should be interpreted as an indicative measure of public sentiment orientation rather than a definitive representation of trust. These findings are therefore temporally bounded and should not be generalized beyond the specific observation period.

The findings reveal that public trust operates along three interrelated layers (affective, cognitive, and structural). Affective trust emerges most visibly in experiential and user-generated platforms, where emotional resonance and peer narratives dominate. Cognitive trust appears more prominently in professional and institutional media, reflecting public evaluation of AI's reliability, ethics, and governance. Structural trust, meanwhile, is rooted in the perceived robustness of digital infrastructure and regulatory frameworks that underpin telemedicine services. Together, these dimensions form a complex ecology of trust, shaped by technology, institutions, and patient experience.

From a methodological perspective, this research demonstrates an integrative application of established computational techniques rather than introducing a fundamentally new methodological advancement. The resulting framework offers a scalable approach for quantifying and interpreting collective attitudes in large-scale, heterogeneous datasets, an essential capability for understanding public sentiment toward emerging health technologies. Conceptually, the proposed *Integrated Model of Public Trust Formation in AI-Enabled Telemedicine* encapsulates the dynamic relationships between technical enablers, institutional forces, and patient-centered experiences, offering a preliminary and interpretive lens for future studies on digital health trust.

In practical terms, the insights presented here inform both policy and design. Policymakers can strengthen public trust by enhancing transparency, ethical communication, and accountability in AI deployment. Healthcare organizations should prioritize user experience and empathy-driven communication, particularly in patient-facing telehealth interfaces. Developers and AI engineers, meanwhile, must focus on explainability, interoperability, and inclusivity to sustain trust at scale. Overall, this study underscores that fostering trust in AI-enabled telemedicine is not solely a technological challenge, it is a sociotechnical imperative requiring alignment between innovation, governance, and human experience.

## Data Availability

The processed data supporting the conclusions of this article will be made available by the authors upon reasonable request.
